# Ascorbic Acid Content and Transcriptional Profiling of Genes Involved in Its Metabolism during Development of Petals, Leaves, and Fruits of Orange (*Citrus sinensis* cv. Valencia Late)

**DOI:** 10.3390/plants10122590

**Published:** 2021-11-26

**Authors:** Enriqueta Alós, Florencia Rey, José Vicente Gil, María Jesús Rodrigo, Lorenzo Zacarias

**Affiliations:** 1 Department of Food Biotechnology, Instituto de Agroquímica y Tecnología de Alimentos, Consejo Superior de Investigaciones Científicas (IATA-CSIC), 46980 Valencia, Spain; enrialos@gmail.com (E.A.); floreyrob@iata.csic.es (F.R.); giljv@iata.csic.es (J.V.G.); mjrodrigo@iata.csic.es (M.J.R.); 2 Food Technology Area, Faculty of Pharmacy, University of Valencia, 46100 Valencia, Spain

**Keywords:** ascorbic acid, *Citrus*, fruit, leaf, maturation, orange, petal, vitamin C

## Abstract

*Citrus* fruit is one of the most important contributors to the ascorbic acid (AsA) intake in humans. Here, we report a comparative analysis of AsA content and transcriptional changes of genes related to its metabolism during development of petals, leaves and fruits of Valencia Late oranges (*Citrus sinensis*). Petals of close flowers and at anthesis contained the highest concentration of AsA. In fruits, AsA content in the flavedo reached a maximum at color break, whereas the pulp accumulated lower levels and experienced minor fluctuations during development. AsA levels in leaves were similar to those in the flavedo at breaker stage. The transcriptional profiling of AsA biosynthetic, degradation, and recycling genes revealed a complex and specific interplay of the different pathways for each tissue. The D-galacturonic acid pathway appeared to be relevant in petals, whereas in leaves the L-galactose pathway (*GGP* and *GME*) also contributed to AsA accumulation. In the flavedo, AsA content was positively correlated with the expression of *GGP* of the L-galactose pathway and negatively with *DHAR1* gene of the recycling pathway. In the pulp, AsA appeared to be mainly controlled by the coordination among the D-galacturonic acid pathway and the *MIOX* and *GalDH* genes. Analysis of the promoters of AsA metabolism genes revealed a number of *cis*-acting elements related to developmental signals, but their functionalities remain to be investigated.

## 1. Introduction

L-ascorbic acid (AsA), also known as vitamin C, is an essential component of the human diet, and fruits and vegetables are the main sources of this compound [1]. Moreover, AsA is a powerful antioxidant that may also decrease the incidence of several illnesses such as cancer or cardiovascular diseases [2]. Hence, the elucidation of AsA accumulation and the factors regulating its concentration in horticultural crops has been a topic of interest, and many studies have focused on assessing AsA concentrations in the edible parts of plants [3,4]. AsA is the most abundant low molecular weight antioxidant present in plant cells and participates in numerous physiological processes, including photosynthesis, growth, and senescence [5]. Due to its notable importance, the enhancement of AsA levels in plants has been carried out by genetic engineering or exogenous application, proving that AsA can mitigate, to some extent, the deleterious effects of environmental stresses in plants [6,7]. In addition, AsA levels have been associated with improved fruit postharvest properties [8,9,10].

**Figure 1:**
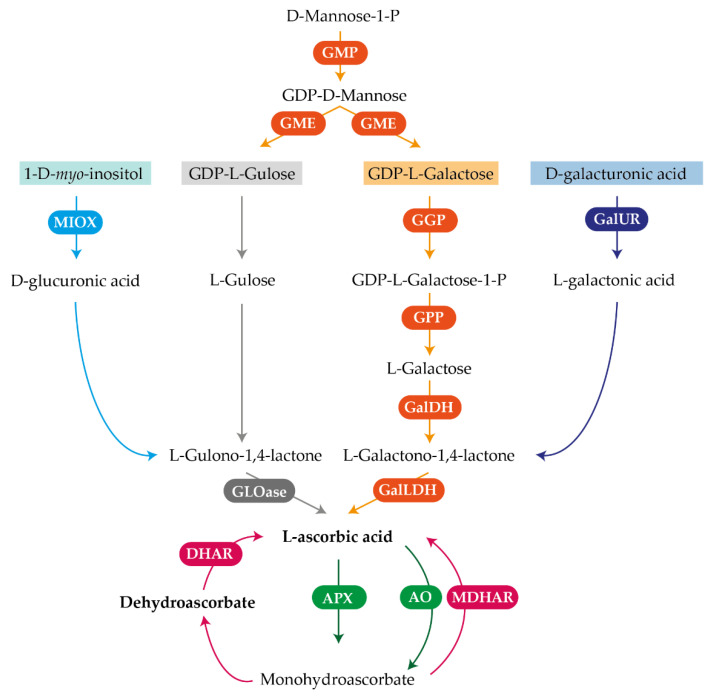
Schematic representation of ascorbic acid (AsA) metabolism, depicting the different biosynthesis pathways and the degradation and recycling pathways in plants. The enzymes catalyzing the reactions are: GDP-mannose pyrophosphorylase (GMP), GDP-mannose-3′-5′-epimerase (GME), GDP-L-galactose transferase (GGP), L-galactose-1-phosphate phosphatase (GPP), L-galactose dehydrogenase (GalDH), L-galactono-1,4-lactone dehydrogenase (GalLDH), D-galacturonic acid reductase (GalUR), *myo*-inositol oxygenase (MIOX), L-gulono-1,4-lactone oxidase (GLOase), monodehydroascorbate reductase (MDHAR), dehydroascorbate reductase (DHAR), ascorbate oxidase (AO), ascorbate peroxidase (APX).

In plants, four possible AsA biosynthetic pathways exist: the L-galactose, L-gulose, *myo*-inositol, and D-galacturonic acid pathways, being the first the principal pathway for AsA biosynthesis in plants [11] (Figure 1). In fruits of many species, the L-galactose pathway also seems to prevail, but the predominance of a particular pathway depends on the species and also varies during fruit development [4]. In line with this, in kiwifruit and immature peach fruits, the L-galactose pathway predominates, whereas in strawberry fruit the D-galacturonic acid and the *myo*-inositol pathways seems to prevail, since expression of different *D-galacturonic acid reductase* isoforms (*GalUR*) and *myo-inositol oxygenase* (*MIOX*) are positively correlated with AsA contents [3]. In tomato, Ioannidi et al. [12] found that most of the genes involved in the L-galactose pathway followed an opposite expression profile to that of AsA accumulation, except for the expression of *GPP* that closely correlated with AsA levels, suggesting a regulatory role of this gene. Moreover, in apple fruit, it has been demonstrated that the de novo synthesis of AsA in the peel occurs through the L-galactose and D-galacturonic acid pathways, while in the flesh and seeds, AsA can only be synthetized by the L-galactose pathway, indicating the AsA biosynthetic pathways also seems to be tissue-specific [13]. In addition to the de novo biosynthesis of AsA, other mechanisms such as the rate of degradation and recycling contribute to the regulation of AsA concentrations in plants (Figure 1). AsA can be transformed into monodehydroascorbate (MDHA) by the enzymes ascorbate oxidase (AO) and ascorbate peroxidase (APX) [14], and the MDHA radical can either be recycled back into AsA by monodehydroascorbate reductase (MDHAR) or undergo disproportionation into dehydroascorbate (DHA) and AsA. Later, DHA can be recycled into AsA by dehydroascorbate reductase (DHAR) before being irrevocably hydrolyzed (Figure 1) [15].

*Citrus* fruits are recognized worldwide as one of the main source of vitamin C for human nutrition, and for centuries it has been known that scurvy—a disease caused by vitamin C deficiency—could be alleviated by eating *Citrus* fruits [16]. Hence, the concentration of AsA in fresh fruits and juices of different species and varieties of *Citrus* has been widely studied [17,18]. However, information about the molecular regulation of AsA metabolism in *Citrus* fruit is still limited. Transcriptional analysis of the genes belonging to the different biosynthetic pathways have been carried out in fruit of orange and mandarin cultivars, which revealed that the L-galactose pathway appears to be the main biosynthetic source in both the flavedo (outer colored part of the peel) and pulp [19,20]. In particular, differences in AsA concentrations among species/cultivars have been associated with differences in the expression of *GDP-mannose pyrophosphorylase* (*GMP*), *GDP-L-galactose transferase* (*GGP*), and L-galactose-1-phosphate phosphatase (*GPP*). Nonetheless, Alós et al. [19] suggested the importance of the D-galacturonic acid and the *myo*-inositol pathways in the regulation of AsA accumulation in *Citrus* fruit tissues, with the genes *D-galacturonic acid reductase12* (*GalUR12*) and *myo*-inositol oxygenase (*MIOX*) playing a key role. More recently, Caruso et al. [21] associated the decrease in AsA content in the juice of blood oranges during maturation to the down-regulation of *MIOX* and *GalUR12*, together with *GDP-mannose-3′*,*5′-epimerase* (*GME*) and *L-Galactose dehydrogenase* (*GalDH*) of the L-galactose pathway. Interestingly, these authors also proposed that the contribution of the different biosynthetic pathways to the AsA pool varied during fruit development, with the L-galactose and *myo*-inositol being important at the early stages of fruit development and the L-gulose pathway at the end of ripening [21]. Moreover, AsA accumulation in *Citrus* fruit seems to be regulated by complex mechanisms affected also by changes in the rate of degradation and recycling [19,20,21]. In particular, differences in APX enzymatic activity have been reported in fruits with contrasting AsA concentrations [20,21], and a marked induction of the *APX2* isoform was observed during development and ripening, suggesting that this enzyme could be crucial for the degradation of AsA in the pulp [19]. In addition, the expression of genes involved in AsA recycling have been correlated with changes in AsA concentrations in the flavedo and pulp during fruit ripening, and also with differences in AsA contents among species or cultivars [19,21].

Furthermore, external factors such as environmental conditions or cultural practices during pre-harvest can also influence the final accumulation of AsA in *Citrus* fruit [22]. In relation to environmental factors, the effect of light on the accumulation and regulation of AsA metabolism has been demonstrated in different *Citrus* species, in which on-tree fruit bagging provoked a reduction of AsA concentrations in the flavedo, without altering the contents in the pulp. Moreover, the absence of light markedly repressed the expression of *GalUR8*, and also *GalUR12* in some species, concomitantly with the reduction of AsA concentrations in the flavedo [23]. These findings support the notion that the D-galacturonic acid plays an important role in AsA accumulation in *Citrus* fruits [19,21]. Information of AsA metabolism in *Citrus* organs other than fruits is very limited and mainly restricted to the analysis of enzymatic activities of AsA degrading and recycling enzymes under stress conditions [24,25,26,27,28]. Nonetheless, the temporal accumulation of AsA in leaves of different orange cultivars has been recently reported [21]. These authors found that AsA contents in leaves varied during the year in a cultivar-dependent manner and suggested that AsA accumulation in vegetative tissue seems to be regulated by the synergistic action of the L-galactose and D-galacturonic pathways [21].

Genetic manipulation of AsA concentrations in different plant species have been successfully achieved by the overexpression of genes encoding AsA biosynthetic and recycling enzymes, or by the suppression of degrading enzymes [3,7,29]. Nonetheless, these transgenic approaches have mainly focused on the whole plant, and results on edible fruits are still limited [7]. In fruits, tomato transgenic lines with altered expression of genes of the L-galactose or D-galacturonic acid have been successful in increasing AsA accumulation, but sometimes with detrimental effects on fruit development [4]. Transcription factors (TFs) have already been identified as key regulators for other metabolic pathways [30], and their manipulation has been more effective for the control of metabolic pathways than using a single (functional) genes [31]. TFs that are directly affecting the expression of genes involved in the AsA metabolic pathways have been described in tomato and Arabidopsis [3]. Moreover, multiple TFs that are co-expressed with genes of the AsA metabolism in tomato fruits have been reported [32]. However, such an approach in other agronomical important crops, as *Citrus*, are scarce. These experimental strategies are required in order to understand the large variability in AsA content and its accumulation pattern among the different species and plant tissues as well as the particular developmental stages of each type of fruit.

Therefore, the aim of the present study was to carry out a detailed comparative analysis of the changes in AsA content and in the transcriptional regulation of its metabolism during development of different organs of oranges (*Citrus sinensis* cv. Valencia Late), namely petals, leaves, flavedo, and pulp of fruit. Additionally, and in order to provide insights on the potential upstream regulators of AsA metabolism in fruits, an analysis of gene co-expression networks and the *cis*-acting elements contained in the promoters of key genes of AsA metabolism has been also accomplished.

## 2. Results

### 2.1. Changes in Ascorbic Acid Content during Development of Petals and Leaves of Valencia Late Oranges

**Figure 2:**
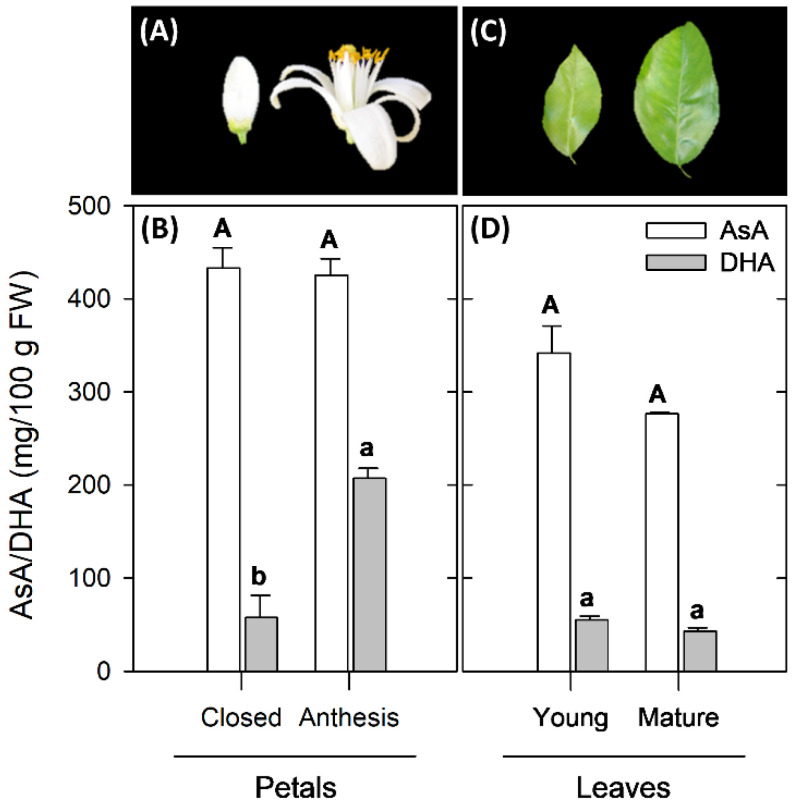
Appearance of flowers (**A**) and leaves (**C**), and ascorbic acid (AsA) and dehydroascorbic acid (DHA) content in petals of closed flowers and flowers at anthesis (**B**), and in young and mature leaves (**D**) of Valencia Late (*Citrus sinensis*) orange. Capital letters indicate significant differences in AsA contents, while lowercase letters indicate significant differences in DHA contents between developmental stages for each tissue (*p* ≤ 0.05, Tukey test). AsA and DHA contents (mg/100 g of FW) are the mean ± S.E. of three replicates.

The AsA and DHA concentration in petals and leaves of Valencia Late at two developmental stages were assessed. Both tissues accumulated high levels of AsA, although contents were higher in petals than in leaves (Figure 2). DHA was detected in both tissues, accounting on average for 22% of total AsA in petals and 13% in leaves. Accumulation of AsA was similar in petals of closed flowers and at anthesis (~430 mg/100 g FW), but DHA increased by nearly 4-times during petal development (Figure 2A,B). It is noteworthy that the concentration in petals was the highest detected in this study. Young-expanding and mature leaves of Valencia orange were selected for AsA analysis (Figure 2C,D). AsA concentration was high in both types of leaves (276–341 mg/100 g FW), and did not experience significant changes during leaf development. Similarly, no variation was detected in DHA content between young and mature leaves (43–55 mg/100 g FW) (Figure 2D).

### 2.2. Changes in Fruit Growth, Peel Coloration, and Ascorbic Acid Content in the Flavedo and Pulp during Fruit Development of Valencia Late Orange

**Figure 3:**
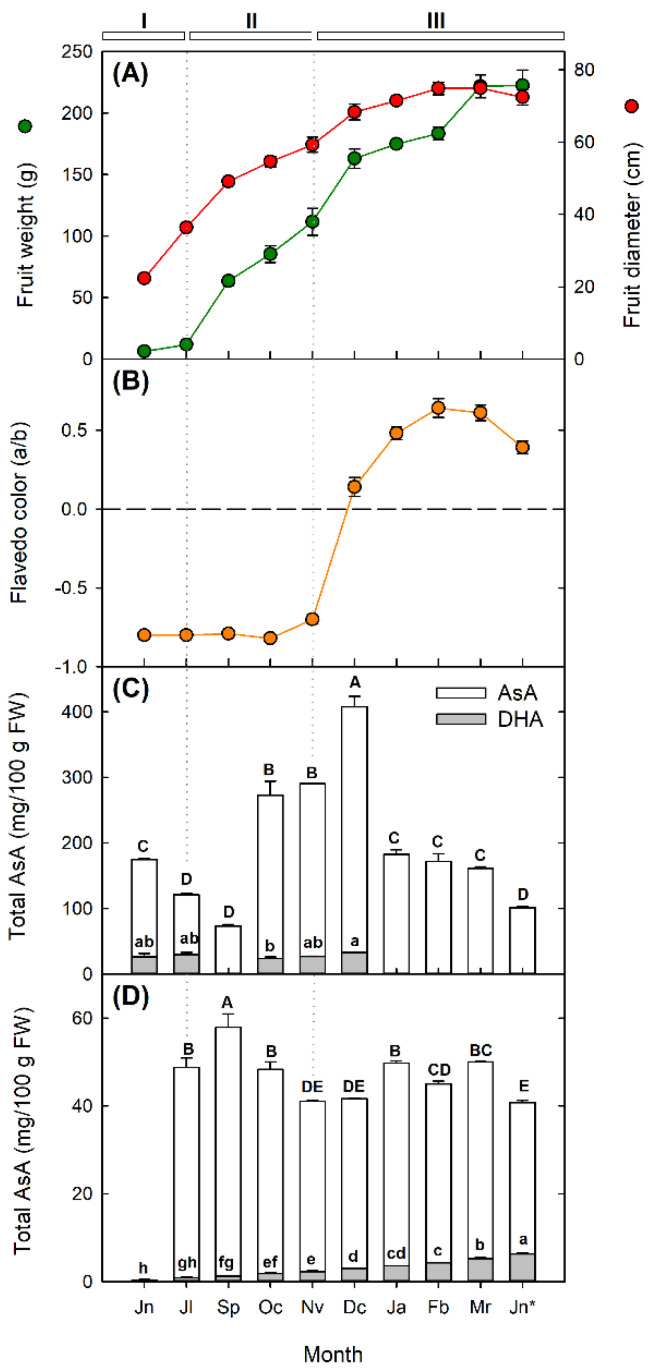
Evolution of fruit weight (green) and diameter (red) (**A**), peel color (**B**) and ascorbic acid (AsA) and dehydroascorbic acid (DHA) contents in the flavedo (**C**) and pulp (**D**) of Valencia Late orange fruit (*Citrus sinensis*) during development and maturation. Fruits were harvested from early development in June (Jn) to over mature fruit in June of the next year (indicated as Jn*). Capital letters indicate significant differences in AsA contents, while lowercase letters indicate significant differences in DHA contents among months for each fruit tissue (*p* ≤ 0.05, Tukey test). Fruit weight (g), diameter (cm), and flavedo color (*a*/*b* Hunter ratio) are the mean ± S.E of 20 fruits, whereas AsA and DHA contents (mg/100 g of FW) are the mean ± S.E of three replicates.

Figure 3A shows the evolution of Valencia Late orange fruit growth and development, expressed as changes in fruit weight and diameter, in which three phases could be distinguished. The first phase of fruit division comprised June and July, in which a slow increase in fruit weight and a significant increment in diameter were observed (Figure 3A). The second phase of fruit development was characterized by a rapid increment in both fruit weight and diameter, and lasted for about 3 months until the peel began color break (November to December). Afterwards, fruit initiated the maturation phase in which the peel color transitioned from green (*a*/*b* < 0) to yellow-orange (*a*/*b* > 0) (Figure 3B), and fruit weight—and to a lower extent fruit diameter—continued to increase.

Important differences in AsA concentration were detected between the flavedo (outer colored part of the fruit peel) and the pulp of Valencia orange fruits, and also in their temporal evolution during fruit development (Figure 3C,D). Contents were always higher in the flavedo than in the pulp, and differences were up to 10-times higher in fruit harvested in December (4.6-times higher in the flavedo, on average of the 10 months analyzed). In the flavedo, total AsA concentrations oscillated between 73 and 407 mg/100 g FW, and three temporal patterns of accumulation could be differentiated, which seemed to be coordinated with the different phases of fruit development. A first wave occurred during the first phase, in which a gradual decrease in AsA contents was detected (from June to September). In the second phase AsA markedly increased (3.4-fold increase from September to October) to reach a maximum in December, coincidentally with color break (Figure 3B). The third wave was characterized by a decline in AsA content in the flavedo of colored fruits (approximately 50% reduction in the flavedo of fruits in January in comparison to December), which remained relatively constant until June, when AsA decreased again in overmature fruits. DHA was only detected in the flavedo of fruits from June until December, and contents remained almost constant or with slight variations (Figure 3C). In the pulp, total AsA was much lower than in the flavedo, with contents varying between 41 to 57 mg/100 g FW (Figure 3D). In immature fruits from June, only traces of AsA were detected, which drastically increased in July. A maximum in AsA content was detected in the pulp of fruits in September, and afterwards contents fluctuated but remained relatively unaltered during fruit development and maturation. DHA levels were detected in the pulp of all the samples analyzed, increasing gradually during development and reaching a maximum in overmature fruit of June (Figure 3D).

### 2.3. Transcriptional Analysis of Genes Involved in the AsA Metabolic Pathways

To understand the transcriptional regulation of the main pathways involved in AsA metabolism during development of petals and leaves, and in the flavedo and pulp of fruit of Valencia oranges, the expression of 23 genes corresponding to AsA key metabolic steps were quantified by qRT-PCR [19]. Genes selected included 13 members from AsA biosynthetic pathways; GDP-mannose pyrophosphorylase (*GMP*), GDP-mannose-3′-5′-epimerase (*GME*), GDP-L-Galactose transferase (*GGP*), L-Galactose-1-phosphate phosphatase (*GPP*), L-Galactose dehydrogenase (*GalDH*), L-galactono-1,4-lactone dehydrogenase (*GalLDH*), *myo*-inositol oxygenase (*MIOX*), and six isoforms of galacturonic acid reductase (*GalUR*); five genes involved in degradation steps: ascorbate oxidase (*AO*) and four isoforms of ascorbate peroxidase (*APX*); and five genes involved in the recycling steps: two isoforms of dehydroascorbate reductase (*DHAR*) and three isoforms of monodehydroascorbate reductase (*MDHAR*) (Figure 1).

#### 2.3.1. Transcriptional Analysis of Genes Involved in AsA Metabolism during Development of Petals and Leaves of Valencia Late Oranges

**Figure 4:**
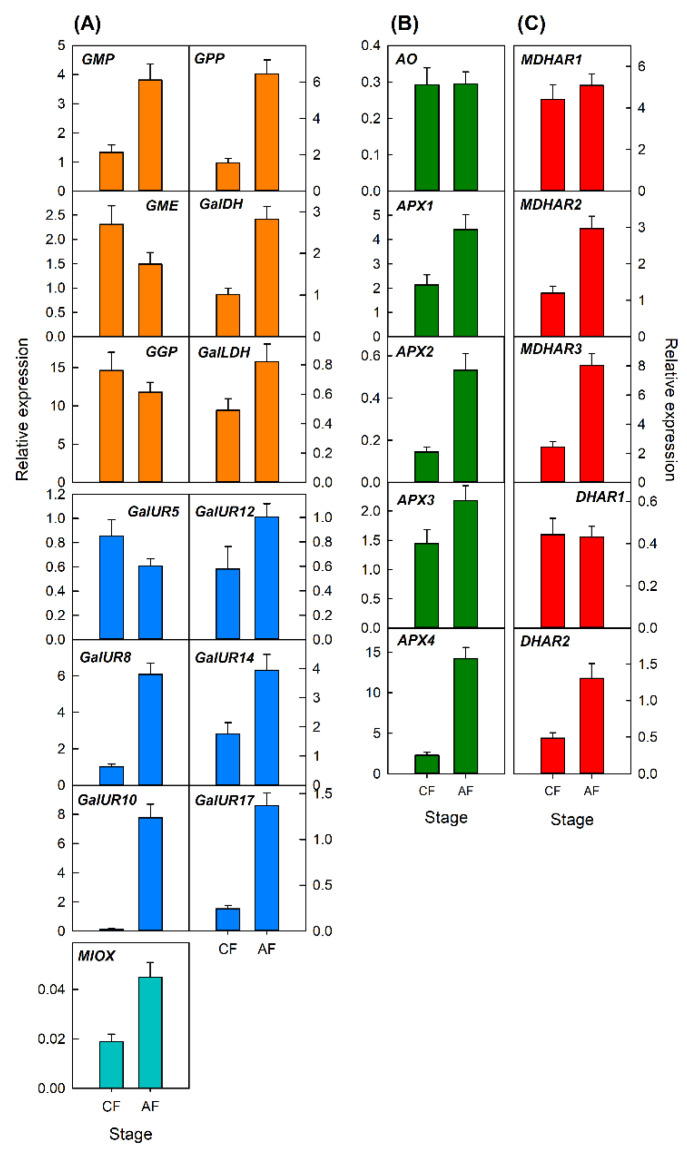
Relative expression of genes involved in the biosynthesis (**A**), degradation (**B**), and recycling (**C**) of AsA in petals of closed flowers (CF) and flowers at anthesis (AF) of Valencia Late orange (*Citrus sinensis*). The genes analyzed were *GMP*, *GME*, *GGP*, *GPP*, *GalDH*, and *GalLDH* of the L-galactose biosynthesis pathway; *GalUR5*, *GalUR8*, *GalUR10*, *GalUR12*, *GalUR14*, and *GalUR17* of the D-galacturonic biosynthesis pathway; *MIOX*, of the *myo*-inositol pathway; *AO*, *APX1*, *APX2*, *APX3*, and *APX4* involved in AsA degradation; and *MDHAR1*, *MDHAR2*, *MDHAR3*, *DHAR1*, and *DHAR2*, involved in AsA recycling. An expression value of 1 was arbitrarily assigned to the values obtained in the flavedo of fruit in June. Results are the mean ± S.E of at least three replicates.

**Figure 5:**
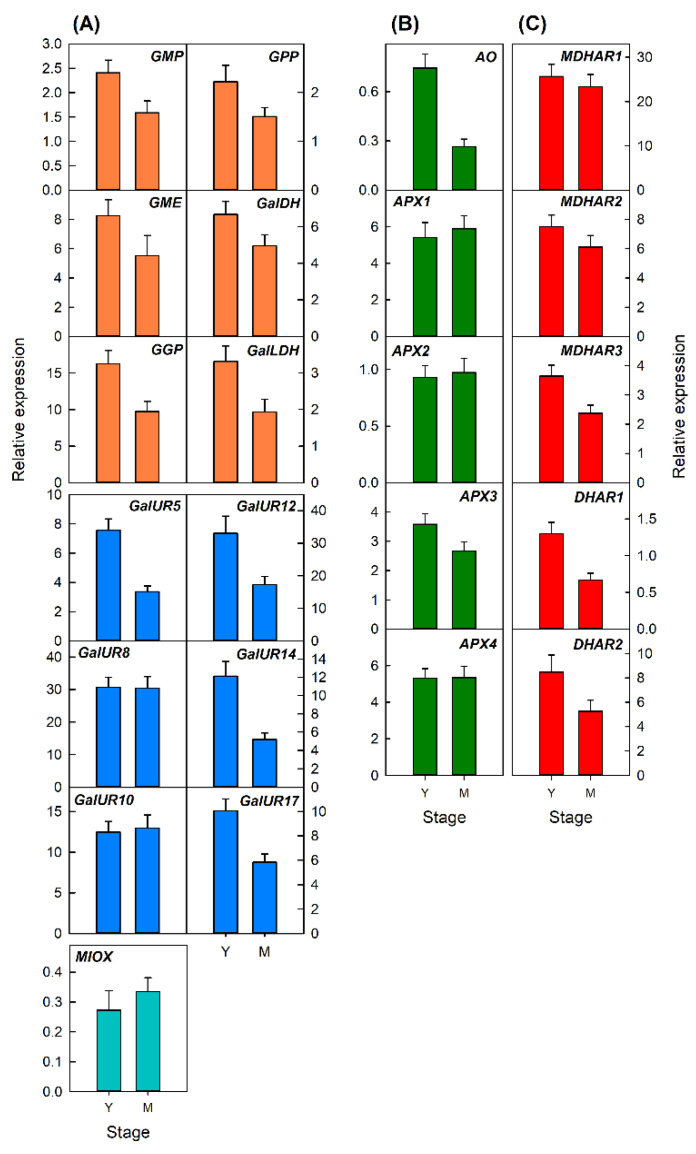
Relative expression of genes involved in the biosynthesis (**A**), degradation (**B**), and recycling (**C**) of AsA in young (Y) and mature (M) leaves of Valencia Late orange (*Citrus sinensis*). The genes analyzed were *GMP*, *GME*, *GGP*, *GPP*, *GalDH*, and *GalLDH* of the L-galactose biosynthesis pathway; *GalUR5*, *GalUR8*, *GalUR10*, *GalUR12*, *GalUR14*, and *GalUR17* of the D-galacturonic biosynthesis pathway; *MIOX*, of the *myo*-inositol pathway; *AO*, *APX1*, *APX2*, *APX3*, and *APX4* involved in AsA degradation; and *MDHAR1*, *MDHAR2*, *MDHAR3*, *DHAR1*, and *DHAR2*, involved in AsA recycling. An expression value of 1 was arbitrarily assigned to the values obtained in the flavedo of fruit in June. Results are the mean ± S.E. of at least three replicates.

Changes in the expression of the selected genes were analyzed in petals and leaves at two developmental stages (Figure 4 and Figure 5). In petals, the expression of most genes increased at anthesis in comparison with those of close flowers (Figure 4). A marked increase (more than 2-fold) was detected in the biosynthetic genes *GMP*, *GPP*, and *GalDH* of the L-galactose pathway, *GalUR8*, *GalUR10*, *GalUR14*, and *GalUR17* of the D-galacturonic acid pathway and *MIOX* of the *myo*-inositol pathway (Figure 4A). The gene *GalLDH* only slightly increased, and a slight decrease was detected for the genes *GME*, *GGP*, and *GalUR5*. With the exception of *AO* which remained at similar levels of expression, the other four genes of the degradation pathway increased at anthesis. Similarly, three genes of the recycling pathway (*MDHAR2*, *MDHAR3*, and *DHAR1*) increased, whereas *MDHAR1* and *DHAR1* did not change during petal development (Figure 4B). These results indicate that the stimulation of AsA biosynthesis during petal development was synchronized with the stimulation of degradation and recycling mechanisms as well.

In leaves, most genes decreased in their relative expression with leaf development (Figure 5). A slight reduction was detected for the genes of the L-galactose pathway, while a marked down-regulation was observed for *GalUR8*, *GalUR10*, *GalUR12*, and *GalUR17* of the D-galacturonic acid pathway (Figure 5A). For the genes involved in AsA degradation, only *AO* and *APX3* were downregulated (Figure 5B), while *MDHAR3*, *DHAR1*, and *DHAR2* of the recycling pathway, were also repressed during leaf development (Figure 5C). It is noteworthy that none of the 23 genes analyzed were stimulated or induced during the development of leaves.

#### 2.3.2. Transcriptional Analysis of Genes Involved in AsA Metabolism in Flavedo and Pulp during Fruit Development of Valencia Late Oranges

**Figure 6:**
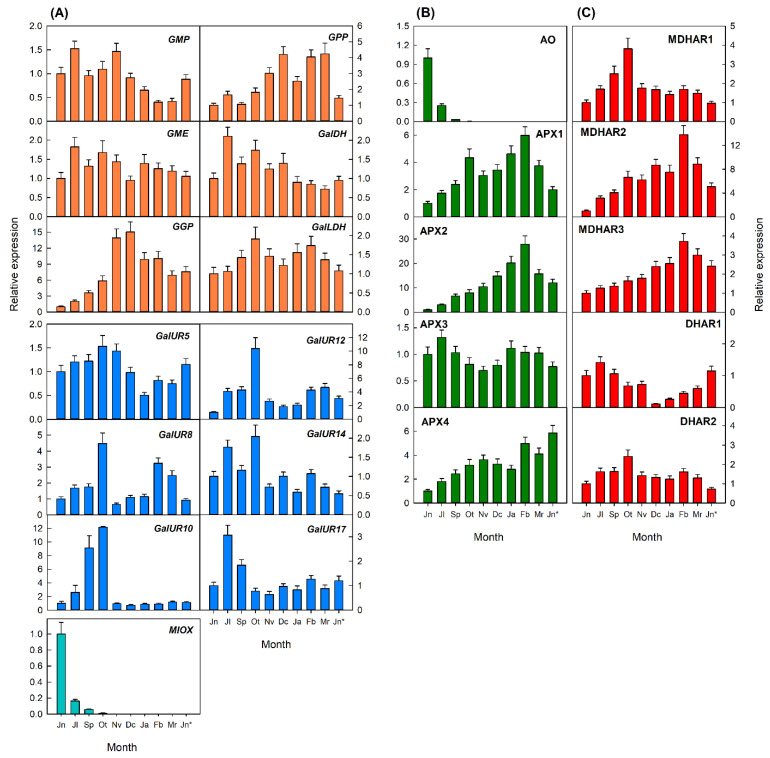
Relative expression of genes involved in the biosynthesis (**A**), degradation (**B**), and recycling (**C**) of AsA in the flavedo of fruit of Valencia Late orange (*Citrus sinensis*) during fruit development and maturation. Fruits were harvested from early development in June (Jn) to overmature fruit in June of the next year (indicated as Jn*). The genes analyzed were *GMP*, *GME*, *GGP*, *GPP*, *GalDH*, and *GalLDH* of the L-galactose biosynthesis pathway; *GalUR5*, *GalUR8*, *GalUR10*, *GalUR12*, *GalUR14*, and *GalUR17* of the D-galacturonic bio-synthesis pathway; *MIOX*, of the *myo*-inositol pathway; *AO*, *APX1*, *APX2*, *APX3*, and *APX4* in-volved in AsA degradation; and *MDHAR1*, *MDHAR2*, *MDHAR3*, *DHAR1*, and *DHAR2*, involved in AsA recycling. An expression value of 1 was arbitrarily assigned to the values obtained in the flavedo of fruit in June. Results are the mean ± S.E of at least three replicates.

**Figure 7:**
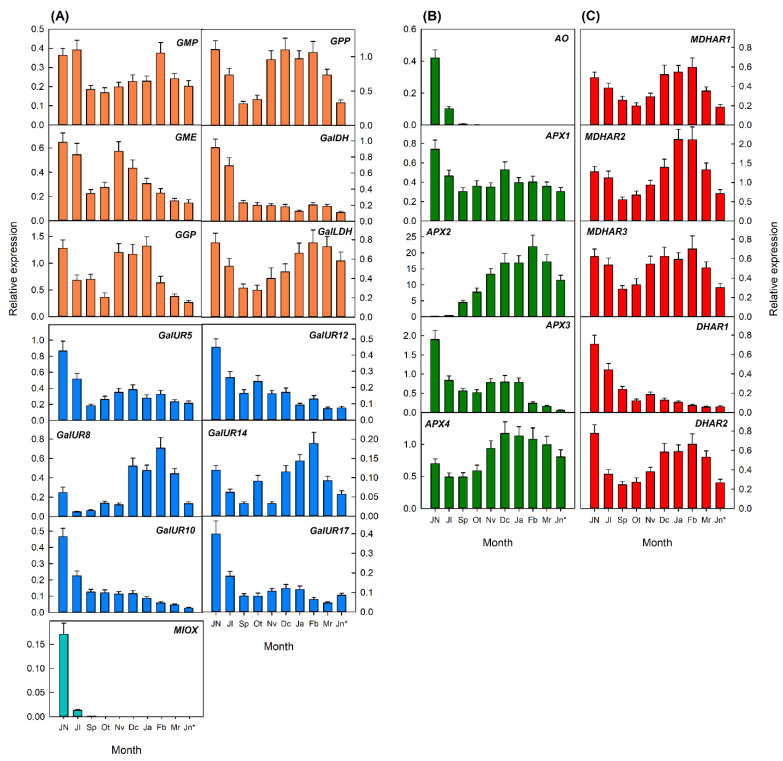
Relative expression of genes involved in the biosynthesis (**A**), degradation (**B**), and recycling (**C**) of AsA in the pulp of fruit of Valencia Late orange (*Citrus sinensis*) during fruit development and maturation. Fruits were harvested from early development in June (Jn) to overmature fruit in June of the next year (indicated as Jn*). The genes analyzed were *GMP*, *GME*, *GGP*, *GPP*, *GalDH*, and *GalLDH* of the L-galactose biosynthesis pathway; *GalUR5*, *GalUR8*, *GalUR10*, *GalUR12*, *GalUR14*, and *GalUR17* of the D-galacturonic bio-synthesis pathway; *MIOX*, of the *myo*-inositol pathway; *AO*, *APX1*, *APX2*, *APX3*, and *APX4* involved in AsA degradation; and *MDHAR1*, *MDHAR2*, *MDHAR3*, *DHAR1*, and *DHAR2* involved in AsA recycling. An expression value of 1 was arbitrarily assigned to the values obtained in the flavedo of fruit in June. Results are the mean ± S.E of at least three replicates.

In fruits, the expression of most genes exhibited a relatively similar temporal profiling in the flavedo and pulp during development, although transcripts levels were in general higher in the flavedo than in the pulp (Figure 6 and Figure 7), in agreement with the differences in AsA concentrations (Figure 3C,D). In the flavedo, the expression of genes of the biosynthetic pathways was very variable. Of the L-galactose pathway, transcripts of *GMP* and *GalDH* were higher at early stages of fruits development and slightly decreased in mature fruits, while *GME* and *GalLDH* showed minor changes in their expression during development and ripening. However, transcript levels of other genes increased after September–October and remained high (*GPP*) or declined (*GGP*) in the flavedo of mature fruits. Interestingly, genes of D-galacturonic pathway were in general more highly expressed during fruit development, and declined at different rates after October or November, coincidentally with the onset of peel color development. Expression of *MIOX* was high in the flavedo of fruitlets form June and markedly decreased during the remaining stages of fruit development and maturation (Figure 6A).

Of the genes involved in AsA degradation, *AO* experienced a marked reduction in July to undetectable levels in the remaining stages of fruit development. The expression of other degradation genes (*APX1*, *APX2*, and *APX4*) increased during development and maturation or experienced slight fluctuations (*APX3*) during the whole process (Figure 6B). Genes from the recycling pathway exhibited two patterns of expression: (i) a gradual increase until mature fruit in February (*MDHAR2* and *MDHAR3*); or (ii) high transcript levels during early development which declined gradually during maturation (*MDHAR1*, *DHAR1*, and *DHAR2*) (Figure 6C).

In the pulp, most transcripts related to the biosynthetic pathways accumulated at high levels in immature fruits and displayed two pattern of expression during development: (i) genes that were downregulated and reached low transcript levels in the pulp of mature fruits (*GalDH*, *GalUR5*, *GalUR10*, *GalUR12*, *GalUR17*, and *MIOX*); and (ii) genes that decreased their expression during fruit enlargement up to November, but increased it again during maturation (January to February) (*GMP*, *GPP*, *GME*, *GGP*, *GalLDH*, *GalUR8*, and *GalUR14*) (Figure 7A). Similarly, genes involved in AsA degradation (Figure 7B) and recycling (Figure 7C) also followed two expression patterns: (i) a downregulation after initial fruit development (*AO*, *APX1*, *APX3*, and *DHAR1*); and (ii) a reduction followed by an enhanced expression to reach high levels in the pulp of mature fruits (December to February) (*MDHAR1*, *MDHAR2*, *MDHAR3*, *APX4*, and *DHAR2*).

Finally, the comparison of the expression of the different genes analyzed in the four tissues of Valencia Late oranges revealed a higher expression of most genes in leaves than in the flavedo, in the flavedo than in petals, and in petals than in the pulp. Genes of the L-galactose biosynthetic pathway and the recycling gene *MDHAR1* were especially stimulated in leaves than in any other tissue, whereas expression of *APX2* was substantially higher in fruit tissues than in leaves or petals. These results suggest that the content of AsA in each orange tissue appears to be coordinated by a complex and specific interplay between the relative expression of genes involved in the biosynthetic and recycling pathways and those involved in the degradation.

### 2.4. Co-Expression Networks of Genes Involved in AsA Metabolism in Flavedo and Pulp of Fruits

**Figure 8:**
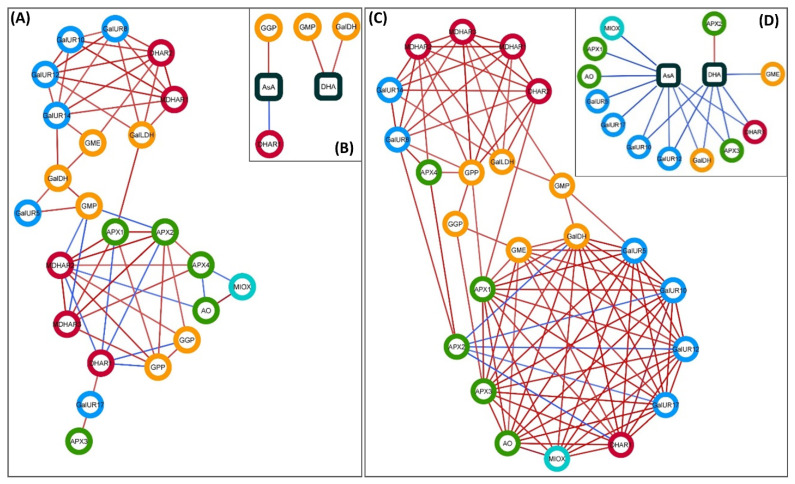
Gene co-expression networks of genes involved in the ascorbic acid (AsA) metabolism in the flavedo (**A**) and pulp (**C**) of Valencia Late orange (*Citrus sinensis*) fruit, and gene-metabolite correlation networks of AsA and DHA contents and the expression of AsA metabolic genes in the flavedo (**B**) and pulp (**D**). In the networks, the color of the circle nodes indicates: yellow, L-galactose biosynthetic genes; blue, D-galacturonic biosynthetic genes; sky-blue, *myo*-inositol biosynthetic genes; green, AsA degradation genes; and red, AsA recycling genes; and the square nodes represent the metabolites AsA, ascorbic acid; and DHA, dehydroascorbic acid. Lines joining the nodes (edges) represent positive correlations (red color) and negative correlations (blue color). The color intensity of the lines represents the strength of the correlation (absolute value of the Pearson’s correlation coefficient). Only significant correlations (*p*-value ≤ 0.05) were taken into account for the construction of the networks. In the gene-metabolite networks (**B**,**D**), only connections between metabolites and genes are represented.

To decipher common regulation mechanisms of genes of the AsA metabolic pathways, a gene co-expression network was built independently for the flavedo and pulp, using the expression data at the different developmental stages (Figure 8A,C). Only significant correlations (Tables S1 and S2) were taken into account to build the networks, in which nodes represented the genes and edges represented positive and negative correlations between them. In addition, a similar analysis was carried out only taking into account the significant correlations between AsA and DHA concentrations and gene expression (Figure 8B,D, Tables S1 and S2). This analysis could not be performed for petals and leaves, since only two developmental stages were analyzed, not allowing a reliable and robust statistical analysis.

Gene co-expression analysis showed that all the genes analyzed (23) were present in both the flavedo and pulp networks, but the number of links between genes was different in the two tissues (Figure 8A,C). Connections in the pulp were higher than in the flavedo, with a media of 8.5 edges in the pulp (98 in total) versus 5.0 in the flavedo (57 in total), indicating that genes seemed to be more co-expressed in the pulp. The architecture of the networks was similar and displayed two main groups with some interrelated genes. In the smaller group, the network comprised almost exclusively of genes belonging to the biosynthetic and recycling pathways, while in the larger group there where genes involved in AsA biosynthesis, recycling, and also degradation. Nonetheless, not all the genes present in the smaller group in the flavedo were present in the same group in the pulp and vice versa.

In the flavedo, 15 genes showed a number of links above the media (*MDHAR2*, *APX2*, *GalUR14*, *DHAR2*, *GPP*, *GalUR8*, *GalUR12*, *APX1*, *MDHAR1*, *MDHAR3*, *DHAR1*, *GMP*, *GalLDH*, *GalUR10*, and *APX4*) with most of them belonging to the degradation and recycling pathways. In the pulp, 11 genes had connections above the media (*GalDH*, *APX1*, *GalUR5*, *GalUR10*, *GalUR12*, *GalUR17*, *APX3*, *DHAR1*, *GME*, *AO*, and *MIOX*), belonging mainly to the biosynthetic pathways. Interestingly, four genes showed a high number of connections in both tissues: *APX1*, *DHAR1*, *GalUR10*, and *GalUR12*.

Another similarity between tissues was that most of the connections among genes in both the flavedo and the pulp were positive, although a higher proportion of total correlations were negative in the flavedo. In particular, the recycling gene *DHAR1* shared a high number of negative links with other genes in the flavedo, indicating a distinct expression pattern of this gene in the flavedo. The expression of this gene was high during the early stages of development and decreased at the beginning of maturation, only to increase again in more mature fruits. In the pulp, *APX2* was the gene with the higher number of negative correlations, mainly to biosynthetic genes but also to genes involved in the degradation of AsA. Interestingly, *DHAR1* was strongly and negatively correlated to *APX2* in both tissues, indicating that these genes have contrasting expression patterns during maturation in both tissues. The largest group of genes interconnected in both tissues was *DHAR2*, *MDHAR1*, *MDHAR2*, *GalUR8*, *GalUR14*, and *GalLDH*, although these last two genes were not correlated with each other in the flavedo. Other associations between two or three genes were found in both tissues, such as *GPP* with *MDHAR2* and *MDHAR3*, or *GPP* with *GGP*, *APX2* with *APX4*, among others.

When the correlations between gene expression and the metabolites AsA and DHA were analyzed (Figure 8B,D), only a few connections between metabolites and genes could be established. In the flavedo, AsA contents were positively correlated to the L-galactose biosynthetic gene *GGP*, indicating that the expression pattern of this gene mirrored the accumulation of AsA in the flavedo during fruit development and maturation (Figure 3C and Figure 6A). By contrast, AsA contents were negatively correlated to *GalUR17* and *DHAR1*, as the expression of these genes was high when contents were low and vice versa. Accumulation of DHA was not correlated to AsA concentrations, and was positively correlated to the expression of *GMP* and *GalDH*. In the pulp, a higher number of connections between metabolites and genes was found than in the flavedo, but most of them were negative. DHA contents were positively linked to the expression of the gene *APX2*, but no positive correlations between AsA contents and the expression of any gene was found. The rest of the correlations between AsA and DHA and genes were negative, and they included mostly biosynthetic genes from the D-galacturonic acid and from the degradation pathway, among others (Figure 8D).

### 2.5. Cis-Acting Regulatory Elements Analysis

Promotor analyses were carried out in order to predict potential transcription factor binding sites (TFBS) involved in the regulation of AsA, by using the software MatInspector (Genomatix software suite version 3.7, Munich, Germany). The MatInspector library of weight matrices for plant TFBS (version 9.4) was screened using 600 bp sequences upstream of the translation start of each of the 23 genes studied. The results were filtered for *p*-value ≤ 0.05, core similarity set to 0.75 and the matrix similarity optimized (Table S3).

**Figure 9:**
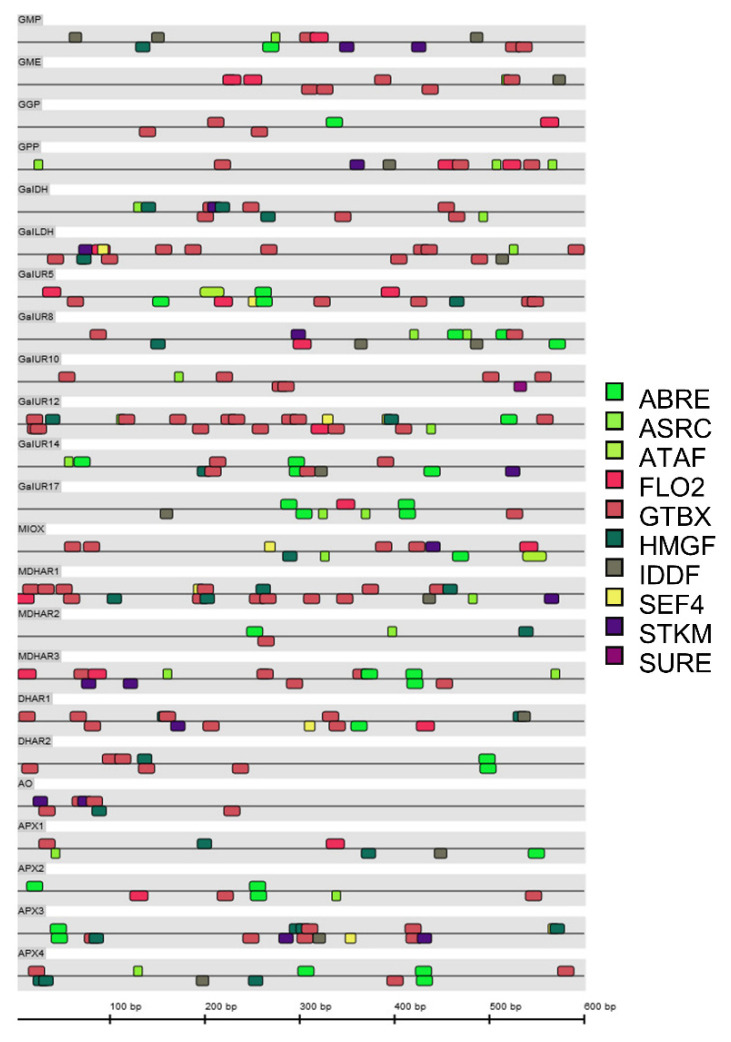
Illustration of transcription factors binding site located in the promoter region of the *Citrus sinensis* genes involved in ascorbic acid (AsA) metabolism. Transcription factors binding sites were obtained comparing the 600bp regions upstream of the start codon (ATG) with the MatInspector database of matrices for plant transcription binding sites (version 9.4), using a *p*-value ≤ 0.05 as cut-off criteria. Abbreviations: ABRE, ABA response elements; ASRC, AS1/AS2 repressor complex; ATAF, ATAF-like NAC domain containing proteins; FLO2, Floral homeotic protein APETALA 2; GTBX, GT-box elements; HMGF, high mobility group factors; IDDF, ID domain factors; SEF4, soybean embryo factor 4; STKM, storekeeper motif; SURE, sugar response elements and SURE-like sequences in plant promoters.

Two hundred and sixty-seven matches were found in the sequences analyzed, which were distributed as follows: approximately 50% of the matches were found in the positive strand and 50% in the negative strand. Ten different putative TFBS were identified in the promotors of at least one of the genes analyzed: ABA response elements (ABRE); AS1/AS2 repressor complex (ASRC); ATAF-like NAC domain containing proteins (ATAF); floral homeotic protein APETALA 2 (FLO2); GT-box elements (GTBX); high mobility group factors (HMGF); ID domain factors (IDDF); soybean embryo factor 4 (SEF4); storekeeper motif (STKM); sugar response elements and SURE and SURE-like sequences in plant promoters (SURE, Figure 9; Tables S3 and S4).

The GTBX putative binding sites were present in all the sequences studied and the number of matches was the highest (112), followed by the ASRC found in 17 genes and, ABRE and the IDDF that were both present in 16 sequences (Table S3). *GalUR12* and *MDHAR1* were the genes with more predicted TFBS, 22 and 21, respectively. In contrast, *GGP*, *MDHAR2*, *GalUR10*, *GalUR17*, *DHAR2*, *AO*, *APX1*, and *APX2* had less than 10 TFBS. The genes *AO*, *DHAR2*, *GalUR10*, and *GGP* contained the lowest number of different DNA binding motifs in the analyzed upstream sequences. The sugar response elements (SURE) were only found in *GalUR10*, while the storekeeper motif (STK), that has sucrose-inducible gene expression, was identified in 12 genes (*GMP*, *GPP*, *GalDH*, *GalLDH*, *GalUR8*, *GalUR14*, *MDHAR1*, *MDHAR3*, *DHAR1*, *AO*, and *APX3* (Figure 9, Tables S3 and S4).

## 3. Discussion

*Citrus* fruit are well known for being excellent sources of vitamin C for humans, and its contents in the pulp and juice of different *Citrus* species has been thoroughly studied [17,33,34]. Although environmental and cultural conditions can affect AsA content in *Citrus* fruit [22], variability appears to be mainly genetically determined and higher AsA contents are usually found in oranges and lemons followed by grapefruits and mandarins [18,33,35]. Moreover, the availability of the *Citrus sinensis* genome sequence [36,37] facilitated the analysis of the transcriptional regulation of many processes in *Citrus*, including AsA metabolism in fruits [19,21,23]. Nonetheless, the molecular regulation of AsA metabolism in other plant organs and tissues of *Citrus* remains poorly understood, and information is mostly limited to the quantification of AsA degrading and recycling enzymes under stress conditions [24,25,26,27,28]. However, seasonal variations in AsA accumulation and transcriptional regulation in leaves have been recently assessed [21]. Thus, the aim of the present work was to shed light on the transcriptional regulation of AsA metabolism in the flavedo and pulp of fruit during the whole reproductive development and also to conduct a comparative analysis in different organs of orange, namely: petals and leaves. To that end, we selected the sweet orange (*Citrus sinensis*) cultivar Valencia Late, which is cultivated worldwide and characterized by a long period of fruit developmental and late ripening, prolonging the harvest time as late as April and May in Northern hemisphere countries.

The analysis of AsA and DHA concentrations revealed that petals were the tissue with the highest levels of total AsA (up to 632 mg/100 g FW at anthesis), which were 1.5-times higher than the maximum detected in leaves and in the fruit flavedo, and 10-times higher than those in the pulp (Figure 2 and Figure 3). Interestingly, petals accumulated not only high amounts of AsA but also important concentrations of DHA. Furthermore, each organ or tissue seemed to have a distinct pattern of accumulation during development. In petals and leaves, AsA remained nearly constant during development, whereas a near 4-fold increase in DHA levels was detected during petal development (Figure 2B,D). In other plant species, AsA content decreased during development of petals of harvested flowers, while it increased or remained constant in flowers remaining in the plant [38,39]. The physiological reason for the high accumulation of vitamin C in petals is not well understood, but it can be reasoned that the short period of petal development during flower expansion and opening may require a high antioxidant status. Moreover, these organs, which usually abscise few days after anthesis, may be a good alternative source of this antioxidant for the industries of cosmetics or dairy supplements, among others.

The concentration of AsA detected in leaves of Valencia oranges revealed that these organs are also rich in AsA and DHA (~400 mg/100 g FW), although with contents lower than petals and comparable to the maximum detected in the flavedo of fruits in December (Figure 3C). The total AsA concentration detected was higher than that reported in leaves of other *Citrus* species, such as Satsuma mandarins (~160 mg/100 g FW) [20], Navel (200–250 mg/100 g FW) or blood orange (<100 mg/100 g FW) [21], but comparable to that found in apple leaves (440 mg/100 g FW) [40]. Interestingly, both AsA and DHA content remained almost unaltered during leaf development, in contrast to the pattern observed in DHA concentration during petal development (Figure 2B). In leaves of other species, such as tea leaves, a similar trend in AsA content with leaf aging was found [41]. Caruso et al. [21] reported important seasonal changes in AsA in leaves of different orange varieties and suggested a tissue and species specificity in the regulation of AsA during development that may explain the differences found in vegetative tissues among the different *Citrus* species.

During the reproductive development of Valencia oranges, differences in AsA concentration and in the pattern of accumulation were detected between the flavedo and pulp, in agreement with previous reports [19,20]. AsA content was higher in the flavedo than in the pulp throughout fruit development, and while it remained relatively constant in the pulp, it experienced large fluctuations in the flavedo (Figure 3C,D). A higher concentration of AsA in the flavedo has previously been reported in different *Citrus* species [19], and also in other fruit species such as apples [13]. It has been suggested that this differential accumulation of AsA may be related to the higher exposure of the flavedo to sunshine during the growing season, as light avoidance dramatically reduced AsA biosynthesis in the flavedo of *Citrus* fruit [23]. Moreover, as the flavedo of the fruit is more widely exposed to stress signals, it is reasonable to assume a larger demand of antioxidants to cope with these adverse conditions. Similarly, other plant antioxidants—such as carotenoids, tocopherols, and flavonoids—have been detected at higher concentrations in the flavedo than in the pulp [42,43,44].

Furthermore, AsA in the flavedo of Valencia oranges changed significantly during development and maturation distinguishing three phases of accumulation (Figure 3C). In the first phase of cell division and early fruit development (form June to September) AsA content declined gradually, suggesting a demand of antioxidants during this period of cell enlargement. Later on, AsA in the flavedo increased to reach a maximum around the end of the second phase (December), coincidentally with the initiation of color break. Afterwards, AsA concentration declined progressively in the flavedo of mature fully colored fruits (Figure 3B,C). Other studies have also detected a maximum of AsA concentrations in the flavedo of fruit of several *Citrus* species during the transition from chloroplast to chromoplasts (October to November), that declined afterwards. Since Valencia Late is a late ripening orange variety with a slow rate of fruit de-greening, it is reasonable that the maximum of AsA content in the flavedo may be reached later than in other varieties, suggesting that the endogenous content of this antioxidant may be species-specific and also influenced by environmental conditions [19,23].

By contrast, AsA concentration in the pulp drastically increased from June to July, and reached a maximum in September. Contents decreased again in October and experienced fluctuations during the remaining months of fruit development and maturation. Although DHA only accounts for a small proportion of total AsA content, concentrations increased gradually during fruit development and reached a maximum in overmature fruit. It is noteworthy the abrupt increase in AsA in the pulp of fruit in July, immediately after the physiological fruit June-drop and when the pulp started to expand and to build up its development (Figure 3D). These results clearly demonstrated a differential regulation of AsA accumulation in the flavedo and pulp of *Citrus* fruits, as it has been reported for other metabolic processes [45]. The content of AsA in the pulp of Valencia Late oranges are in a similar range to those reported in the juice of Navel and blood oranges, but in these varieties, it decreased more remarkably towards fruit maturation [21]. This different pattern of accumulation may be related to the different plant material, juice vs. pulp, but also to genotypic differences [20,21]. In fruits of other species, such as tomato [12] and pepper [46], AsA increased with maturation, whereas in apple, cherry, and peach it was higher at immature stages [47,48,49].

Transcriptional analysis of the main genes involved in AsA metabolism revealed that AsA accumulation in the different organs and tissues is regulated in a complex and specific manner, and seems to depend on the relative balance between the rate of biosynthesis and the mechanisms of AsA degradation and recycling (Figure 1) [3,4]. In petals, the changes in AsA and DHA contents were accompanied by the upregulation of most biosynthetic and recycling genes, but also of those involved in degradation (Figure 4). A substantial induction (more than 4-times) was detected in the genes *GPP* and some *GalUR* isoforms, indicating the importance of the D-galacturonic pathway in the biosynthesis of AsA in this organ. Expression of *GPP* has been correlated to AsA levels in different plant tissues, and is considered a pivotal step in AsA synthesis [3]. Interestingly, AsA contents during petal development remained constant and the increase was mainly in DHA (Figure 2B). Therefore, it is possible that the relative contribution of AsA degradation is favored over the recycling, probably by the stimulation of the expression of all the *APX* isoforms and the low rate of DHA conversion to AsA (Figure 4B,C). Collectively, these results indicate that in petals of orange, a synchronized activation of the different pathways during flower opening is playing a major role in the maintenance of AsA homeostasis.

In the leaves, the evolution of AsA during development was accompanied by a downregulation in the majority of the genes analyzed (Figure 5). All genes involved in the L-galactose biosynthesis pathway and most of the *GalUR* isoforms were repressed during leaf development (Figure 5A). These results are in agreement with those of Caruso et al. [21] in leaves of Navel and blood oranges, suggesting the synergistic contribution of the L-galactose and D-galacturonic pathways to AsA synthesis in *Citrus* leaves. Moreover, the expression of most AsA degradation genes was maintained (*APX1*, *APX2*, *APX4*), while most of the recycling genes were downregulated. The fact that the downregulation of *AO* and *APX3* did not produce an increase in AsA suggest, overall, a coordinated decrease in the expression of biosynthetic and recycling genes to maintain AsA accumulation.

Significant differences in the transcriptional regulation of AsA were also detected between flavedo and pulp of Valencia orange fruits. In the flavedo, accumulation of AsA positively correlated with the expression of *GGP* of the L-galactose pathway (Figure 6 and Figure 8B). Expression of this gene increased from June to November–December when AsA contents were maximum in the flavedo, and then declined towards maturation in agreement with the decrease in contents (Figure 3C and Figure 6A). The induction of *GPP* after November may also help to maintain the synthesis of AsA at later stages of maturation (up to February). Moreover, the expression of *GMP* and *GalDH* positively correlated to DHA contents (Figure 8B). It has been previously indicated that AsA accumulation in the flavedo, and also in the pulp and juice, is correlated with the transcriptional profiling of some of the L-galactose pathway genes [19,20,21], and in particular with the expression of *GGP* [19]. These results reinforce the notion that *GGP* is an important regulatory step of AsA content in *Citrus* flavedo. Similarly, AsA levels have been correlated to *GGP* and *GPP* transcript levels in tomato fruit [12,50] and *GGP* in apple [51]. Furthermore, it is known that AsA concentrations exert a negative feedback on the expression of *GGP* [52], and this could explain the decrease in transcript accumulation detected in the flavedo of Valencia fruits after reaching the maximum of AsA content in December (Figure 3C and Figure 6A).

Beside the relevance of the L-galactose pathway, the elevated expression of *MIOX* in young fruitlets (June)—which drastically decreased towards October—suggest the involvement of this pathway in determining AsA content at the early fruit development stages of Valencia Late oranges (Figure 3C and Figure 6A). In Washington Navel oranges, the involvement of *MIOX* at early stages of fruit development has been also proposed [19]. Thus, it seems that the *myo*-inositol pathway may participate in the synthesis of AsA at the initial stages of fruit development, followed by a major involvement of the L-galactose and D-galacturonic pathway during the last phases of cell enlargement and during the whole period of fruit maturation. In addition to these transcriptional changes, the correlation network of the flavedo of Valencia oranges revealed a negative correlation of AsA content with the *DHAR1* gene (Figure 8B). Accumulation of the transcript of this gene reached the lowest levels in December (Figure 5C), coincident with the maximum of AsA content (Figure 3C). Thus, it is likely that the expression of *DHAR1* in coordination with other genes involved in AsA degradation may be part of the mechanisms to maintain the homeostasis of the AsA pool in the flavedo of fruits.

Regarding the transcriptional regulation of AsA in the pulp, the correlation network revealed that most of the biosynthetic genes of the D-galacturonic pathway (*GalUR5*, *GalUR10*, *GalUR12*, and *GalUR17*), and also *MIOX* and *GalDH*, were negatively correlated with AsA concentration. Moreover, *GalUR12*, *GalUR17*, *GalDH*, and *GME* were also negatively correlated with DHA content (Figure 8D). The fact that most of these genes were downregulated, whereas AsA experienced slight variations suggest that the transcriptional regulation of these pathways is not playing a relevant role in AsA accumulation. Other genes of AsA degradation (*APX1*, *APX3*, and *AO*) were also negatively related to AsA content, contributing also to the maintenance of the homeostasis. Finally, a close examination of the expression of most genes of the L-galactose and the recycling pathways, as well as *APX2* and *APX4* of the degradation pathway, revealed a similar transcriptional profiling (Figure 7). Taken together, these results indicate that the regulation of AsA content in the pulp of Valencia orange fruits may be the result of a complex and coordinated balance between biosynthetic, degradation, and recycling pathways, contributing to keeping the content of this antioxidant relatively stable during fruit development and maturation.

Analysis of the co-expression of genes of the AsA metabolic pathways and the search of *cis*-elements in the promoter region of these genes can help to decipher common regulators of the process and to understand the mechanisms and environmental signals involved in the synthesis and accumulation of AsA. Up to now, there are only few TFs known to affect either positively or negatively AsA metabolisms in plants, as HDZIP1 in tomato fruits [32], and ERF98 and AMR1 in Arabidopsis [53], but none has been so far described in *Citrus*.

The in silico analysis of the promoter regions of the 23 genes of AsA metabolism analyzed revealed interesting features (Figure 9). GT factor binding motifs are present in all the genes analyzed. These motifs have been found in the promoters of a number of plant genes, but their regulatory function of these TFs remains unknown [54]. Moreover, ABA and sugar responsive elements (ABRE, STK, and SURE) where found in the majority of the genes, indicating that the regulation of AsA metabolism may be mediated by this phytohormone and sugars. This is an interesting finding supported by previous data, since evidence of the overlapping among ABA, ethylene, and sugar signaling in the modulation of the redox status of the cells have been reported in Arabidopsis [55,56,57]. Moreover, ABA treatments induced the expression of *DHAR1* and *DHAR2* in pepper leaves [58] and the promoter of Arabidopsis *APX2* gene contains ABA response elements [59]. Additionally, ABA treatment increased AsA content in raspberries [60] and in strawberry also stimulated the expression of genes involved in its biosynthesis [61]. The presence of ABA-responsible elements in the promotor region of many genes related to AsA metabolism in Valencia Late orange suggest a potential influence of this hormone in the regulation of AsA content. This suggestion is not unexpected, since it is well documented that ABA concentration increased in the flavedo of orange fruit during color break [62], and it is likely that this may participate in the mechanisms controlling AsA accumulation in this tissue.

Ascorbic acid is known to modulate plant growth processes including flowering [6,63,64]. Hence, the fact that APETALA2 (AP2) binding motifs are found in genes involved in AsA metabolism could indicate a plausible link for this relationship (Tables S3 and S4), especially taking into account that AP2 is involved in a wide variety of developmental processes [5]. In tomato, NAC TFs were co-expressed with AsA metabolic genes and in our analysis the binding motifs to ATAF-2, which is a NAC domain containing protein, were found in *GalUR5* and *MIOX* [65]. Even the presence of these regulatory signatures in the promotor of genes related to AsA metabolism does not allow us to yet explain their transcriptional pattern of expression in the different organs, and tissues of Valencia Late oranges are illustrative of the myriad of endogenous factors and environmental signals that may regulate AsA content in a tissue-specific manner.

## 4. Materials and Methods

### 4.1. Plant Material and Growth Conditions

All the plant material (petals, leaves, and fruits) used in this study were harvested from adult trees of oranges (*Citrus sinensis* L., cv. Valencia Late) grafted on Citrange carrizo rootstock (*Citrus sinensis* × *Poncirus trifoliata*) grown at the Citrus Germplasm Bank of the Instituto Valenciano de Investigaciones Agrarias (+39°35′22″, −0°23′40″, Moncada, Valencia, Spain). Leaves were collected at two developmental stages: young developing leaf (Y) and mature about 12 months old (M). Petals were carefully collected from closed flowers (CF) and at anthesis (AF). Fruits were harvested at 10 developmental stages: from small fruitlets (June and July) collected after the physiological June-drop to full mature fruits (March) and over-mature fruits (about 14-months after flowering, indicated as June*). Immediately after harvest, the plant organs were delivered to the laboratory and selected for uniformity and the absence of any lesion or injury. The fruit equatorial diameter was measured in 20 fruits with a manual caliper, and the same fruits were used to determine fruit weight during development. Peel color of the same fruits was measured using a Minolta CR-330 colorimeter (Minolta, Osaka, Japan) on three locations around the equatorial plane of the fruit. Color index was expressed as the *a*/*b* Hunter ratio [66,67], which is negative for green fruit, around zero for yellow fruit at color break, and positive for orange colored fruit. The flavedo (outer colored part of the fruit peel) and pulp of fruits at the different development stages were separated with a scalpel. All plant tissues were frozen in liquid nitrogen, ground to a fine powder and stored at −80 °C until analysis.

### 4.2. Ascorbic Acid Extraction and Analysis by HPLC

Ascorbic acid was extracted and determined essentially as described in Alós et al. [46]. Briefly, 0.5 g of tissue was homogenized for 1 min using a Polytron homogenizer (Polytron, Kinematica, Eschbach, Germany) with 0.1% metaphosphoric acid (4 mL). The homogenate was then centrifuged for 10 min at 4500× *g* at 4 °C. The supernatant was passed through a C_18_ cartridge (SepPak, Waters, Barcelona, Spain), previously activated with 4 mL of methanol, 4 mL of water and 4 mL of 2% metaphosphoric acid. The extract was subsequently filtered through a 0.45 μm nylon filter (25 mm diameter, Análisis Vínicos, Tomelloso, Spain). This filtrate was directly used for AsA determination, while a 500 μL aliquot of the same filtrate was incubated for one hour in the dark with 5 μL of DTT (500 mM) for the determination of total vitamin C. Determination of total vitamin C content and AsA was carried out using a Dionex HPLC system with a photodiode array detector (PDA) and Chromeleon software (Dionex, Thermo Fisher Scientific, Barcelona, Spain). An Ultrabase C_18_ column (100 × 4.6 mm, 2.5 μm) and a mobile phase of methanol: water pH 2.5 (adjusted with metaphosphoric acid, 15:85, *v*/*v*), 0.2 mL min^−1^ flux and injection volume 10 μL. The temperature of the column was set at 35 °C. The method was calibrated with a curve of an ascorbic acid standard solution in 2% metaphosphoric acid, with concentrations between 1 and 100 μg/mL. Dehydroascorbic acid content was calculated as the difference between total vitamin C and AsA contents.

### 4.3. RNA Extraction and cDNA Synthesis

Total RNA was isolated from the fruit tissues using RNeasy Plant Mini Kit (Qiagen, Madrid, Spain) and subsequently treated with DNase I (DNA free, DNase treatment & removal, Ambion, Barcelona, Spain). The amount of RNA was measured by spectrophotometric analysis (Nanodrop, Thermo Fisher Scientific, Barcelona, Spain) and its quality was verified by agarose gel electrophoresis with ethidium bromide staining. The absence of DNA contamination was checked by performing a no-reverse transcription assay which consisted of a PCR with each RNA sample using the ACTIN primers [19]. No amplified products were detected which confirmed the purity of the RNA extracts. The transcripts present in 5 μg of total RNA were reverse-transcribed using the SuperScript III Reverse Transcriptase (Invitrogen Barcelona, Spain) in a total volume of 20 μL. One μL of a 10-fold diluted first-strand cDNA was used for each amplification reaction.

### 4.4. Gene Expression Analysis by Real Time PCR

Quantitative real-time PCR was carried out on a LightCycler 480 instrument (Roche, Madrid, Spain), using the LightCycler 480 SYBRGreen I Master kit (Roche, Madrid, Spain). Reaction mix and conditions followed the manufacturer’s instructions with some modifications. The PCR mix contained 1 μL of diluted cDNA, 5 μL of SYBR Green I Master Mix, 1 μL of 3 μM primer F, and 1 μL of 3 μM primer R, with a final volume of 10 μL. The primers (PSF purified, Isogen, Utrecht, Netherlands) used for the amplification of each gene are described by Alós et al. [19]. The cycling protocol, for all genes, consisted of 10 min at 95 °C for pre-incubation, then 40 cycles of 10 s at 95 °C for denaturation, 10 s at 59 °C for annealing, and 10 s at 72 °C for extension. Fluorescent intensity data was acquired during the extension time with the LightCycler 480 Software release 1.5.0, version 1.5.0.39 (Roche, Madrid, Spain) and were transformed into mRNA levels by using specific standard curves for all analyzed genes. Amplification efficiency (E) and correlation coefficient (*r*^2^) of each primer were calculated using the standard curve method and the formula E = 10^(^^−^^1/slope)^ [68].

The specificity of the PCR reaction was assessed by the presence of a single peak in the dissociation curve performed after the amplification steps followed by the sequencing of the amplicon. In this experiment *ACTIN*, *ELONGATION FACTOR 1* (*EF1*), and *β-TUBULIN* (*β-TUB*) were tested as potential housekeeping genes based on previously published primer sequences of *Citrus* genes using BestKeeper software [69]. The *ACTIN* gene was the best housekeeping gene for our analysis, having the lowest SD [±CP], therefore the normalization of the gene expression levels was done against it. Expression levels were calculated using the Relative Expression Software Tool (REST, [70]) and relative to the expression value obtained for each gene in the flavedo of immature-green Valencia Late fruits (collected in June), which was arbitrarily given the expression value of 1. Results are the mean of at least three replicates.

### 4.5. Search for Putative Transcription Factor Binding Sites in the Promoter Region of Genes Involved in AsA Metabolism

The DNA sequences of the promoter regions (600 bp upstream of the start codon ATG) were obtained for the 23 genes involved in AsA metabolism analyzed in this work. Sequences were downloaded from the *Citrus sinensis* (sweet orange) genome databases Huazhong Agricultural University (http://citrus.hzau.edu.cn/orange, accessed on 14 April 2021; [36]) and Phytozome (JGI Phytozome v12; [37]), and are available in Table S5. Subsequently, the promoter regions were used to carry out a screening analysis in the MatInspector library of matrices for plant transcription binding sites version 9.4 (Genomatix software suite v3.7, Munich, Germany, [71,72]) Only the matrices with a *p*-value equal or lower than 0.05 were considered in the present work. The *p*-value represents the probability to obtain an equal or greater number of sequences with a match in a randomly drawn sample of the same size as the input sequence set, hence the lower this probability, the higher is the importance of the observed TFBS.

### 4.6. Co-Expression Gene Networks

To highlight possible co-expression of genes during fruit development and maturation, and also possible correlations between AsA and DHA concentrations and genes, two separate correlation networks were built for the fruit flavedo and pulp: (i) a gene co-regulation network representing only correlations between genes; and (ii) a network showing only correlations between genes and metabolites (AsA and DHA). For the construction of the networks, a Pearson’s correlation analysis was first carried out for each fruit tissue, taking into account all the stages of fruit development and maturation. The input data for the calculation of correlation coefficients (*r*^2^) was the fold-change relative to the values obtained in the flavedo or pulp, respectively, at the immature green stage (June) for each gene (relative expression levels) or metabolite (AsA and DHA concentrations). The *p*-values for the correlation of each gene–gene or metabolite–gene pair were also calculated. Correlation analyses were carried out in RStudio (version 1.3.1093, RStudio Team, PBC, Boston, MA, USA) using the function “cormat”. Networks were built considering only significant correlations (cutoff criteria: *p*-value ≤ 0.05), and assembled manually using the software Cytoscape (version 3.8.2, National Institute of General Medical Sciences, Bethesda, MD, USA). Genes and metabolites correspond to the network nodes, which are linked by lines (edges) if a significant correlation exists between two genes or between a gene and metabolite. Positive correlations are shown in red, while negative correlations are in blue, and the color intensity represents the strength of the correlation (absolute value of Pearson’s correlation coefficient). Networks were not constructed for petals and leaves data, as not enough dates were registered to construct a reliable correlation network.

### 4.7. Statistical Analysis

To determine if differences in means were significant among the developmental stages analyzed for each tissue, data for AsA and DHA content was subjected to a one-way analysis of variance (ANOVA) followed by Tukey’s test (significance level at *p* ≤ 0.05), using the InfoStat software (version 2018, Grupo Infostat, Córdoba, Argentina).

## Data Availability

The data presented in this study are available in article and Supplementary Material.

## Supplementary Materials

The following are available online at https://www.mdpi.com/article/10.3390/plants10122590/s1, Table S1. Correlation matrix analysis of metabolite levels and gene expression in the flavedo of Valencia Late orange fruit (*Citrus sinensis*) during fruit development and maturation; Table S2. Correlation matrix analysis of metabolite levels and gene expression in the pulp of Valencia Late orange fruit (*Citrus sinensis*) during fruit development and maturation; Table S3. List of putative transcription factor binding sites identified in the promoter region of 23 genes involved in AsA metabolism in *Citrus sinensis*, obtained by comparison with the matrices of the MatInspector database version 9.4 (Genomatix software suite version 3.7). Total matches indicate the number of matches found in all input sequences and common to a number of genes means the number of sequences where this transcription factor binding sites were found; Table S4. Detailed information about the putative transcription factor binding sites identified (*p*-value ≤ 0.05) in the promoter regions of 23 genes involved in AsA metabolism in *Citrus sinensis*; Table S5. Sequences used in the search of the putative transcription factor binding sites. These sequences were downloaded from the genome of sweet orange (*Citrus sinensis*) from the Huazhong Agricultural University (http://citrus.hzau.edu.cn/orange, accessed on 14 April 2021; [36]) and Phytozome (JGI Phytozome v12; [37]) databases.
